# Determinants of Left Ventricular Global Longitudinal Strain in a Ghanaian Cohort

**DOI:** 10.7759/cureus.99118

**Published:** 2025-12-13

**Authors:** Andrew S Dzebu, Sheila Attuquayefio, John Kpodonu

**Affiliations:** 1 Cardiothoracic Center, Ho Teaching Hospital, Ho, GHA; 2 Cardiology, University of Ghana Medical Center, Accra, GHA; 3 Department of Medicine and Therapeutics, University of Ghana Medical School, Accra, GHA

**Keywords:** echocardiography, heart failure, left ventricular global longitudinal strain, speckle tracking echocardiography, strain imaging

## Abstract

Background

Cardiovascular disease, particularly heart failure, poses a significant global health burden, especially in low- to middle-income regions like Africa, where resources are limited. Heart failure is often related to various risk factors. Modern imaging techniques, like speckle-tracking echocardiography, enhance the detection of subclinical cardiac dysfunction. Early identification and management of these risk factors could prevent progression to HF, emphasizing the need for routine, patient-level interventions.

Objectives

The specific objective was to find the correlation between the clinical risk factors and conventional echocardiographic/Doppler markers of diastolic and systolic dysfunction with left ventricular global longitudinal strain.

Methods

This was a single-center, retrospective, analytical study of consecutive patients who underwent echocardiograms, in which we compared demographic, clinical, and 2-dimensional echocardiographic variables of diastolic and systolic function with left ventricular global longitudinal strain using univariate linear regression analysis. This study received ethical clearance with Approval No. HTH-REC(38) FC_2024 from the Ho Teaching Hospital Research and Ethics Committee (HTH-REC).

Results

The average patient age was 57 years. About half were female patients; most were overweight or obese, and had blood pressures in the hypertensive range. Most had normal two-dimensional echocardiographic parameters, but with borderline or abnormal left ventricular global longitudinal strain. Body mass index, systolic and diastolic blood pressure, left ventricular mass index, e’, E/e', and left ventricular ejection fraction correlated significantly with left ventricular global longitudinal strain.

Conclusion

The burden of traditional risk factors for heart failure and structural/functional cardiac abnormalities is high among patients who presented to a community cardiologic clinic. Body mass index, systolic blood pressure, diastolic blood pressure, e’, E/e’, and left ventricular ejection fraction (LVEF) independently correlated with left ventricular global longitudinal strain (LVGLS), a valuable parameter in evaluating left ventricular (LV) systolic function, even when LVEF is normal, underscoring its role in risk stratification and early intervention for patients at risk of heart failure.

## Introduction

The global burden of cardiovascular disease (CVD) is high [1]. Heart failure (HF) represents a late-stage confluence of many etiologies of cardiovascular disease. It is a major healthcare issue characterized by high resource utilization and increased healthcare costs. Incidence is generally stable, but prevalence is rising due to longer life expectancy enabled by more effective treatments [2].

There is a high burden of cardiovascular risk factors in low -to middle-income countries such as Ghana [3-6]. However, this region is unprepared due to a disproportionate allocation of resources, including an inadequate number of cardiovascular specialists. Unlike the rest of the world, there are few population-based heart failure studies in Africa, compared to hospital-based studies. Hence, a more precise description of this public health concern is limited [2].

Risk factors such as hypertension, diabetes mellitus, obesity, dyslipidemia, and tobacco use, among others, have been known to precede the onset of cardiovascular diseases, especially atherosclerotic cardiovascular disease. Risk factors may also be identified using non-invasive cardiovascular imaging techniques, such as two-dimensional echocardiography, tissue Doppler imaging, and speckle tracking echocardiography. The presence of a risk factor may indicate subclinical cardiac dysfunction in the absence of overt heart failure [7-10]. Clinical heart failure is preceded by structural and functional cardiac abnormalities, many of which are diagnosable using imaging modalities such as echocardiography. The presence of these cardiac abnormalities in an asymptomatic person is considered pre-heart failure (stage B heart failure) [10,11].

Timely intervention to adequately address these risk factors may delay or prevent progression to clinical heart failure. A selective or systematic search for these factors should be incorporated into routine clinical practice. It has been suggested that interventions targeting risk factors at the population level should substantially reduce the burden of overt cardiovascular disease [11-13].

Speckle tracking echocardiography is a relatively new imaging technique compared to conventional echocardiography. The most common variant applied in clinical practice is left ventricular global longitudinal strain (LVGLS), which has diagnostic importance and can detect subclinical systolic dysfunction, even when left ventricular ejection fraction (LVEF) is preserved, as can occur in myocardial disease, in cancer therapy-related cardiac dysfunction. It is also used in the differential diagnosis of left ventricular hypertrophy (e.g., hypertensive heart disease, infiltrative disease, hypertrophic cardiomyopathy, aortic stenosis, etc.), in the detection of regional strain abnormalities associated with coronary artery disease, and in other conditions. LVGLS is additionally helpful for prognostication. It also addresses some challenges related to conventional echocardiographic parameters, such as LVEF (geometric assumptions, wide intra- and inter-observer variability, etc.), and is complementary to conventional echocardiography [14-16].

Determinants of LVGLS include demographic and anthropometric factors (e.g., age, sex, body mass index, race), hemodynamic factors (e.g., preload, contractility, afterload, heart rate), geometric factors (e.g., left ventricular geometry), and myocardial tissue characteristics. These determinants may increase or decrease left ventricular longitudinal strain, even in the absence of myocardial disease per se [16-18].

Given the scanty literature on strain imaging in Ghana and the general African context, and with significant disparity when compared to high-income countries [19], we explore LVGLS in patients who reported for echocardiography in a community cardiovascular practice, assessing their relations with clinical and traditional echocardiographic parameters related to heart failure.

To the best of our knowledge, local normative data have not been published. The novelty of our study lies in the strain data from West Africa, which may be useful for comparative studies with data from other regions. It also highlights the need for normative data on strain and other echocardiographic parameters, which may help formulate chamber quantification guidelines if local data differ considerably from those in other regions of the world.

## Materials and methods

Study design and population

This was a single-center retrospective analytical study of consecutive patients who underwent echocardiograms. The data were obtained from the echocardiography archiving system used at Luccahealth Medical Specialty Center in Accra, Ghana. Approval was provided by the Ethics and Research Committee of the Ho Teaching Hospital, Ghana (Approval No. HTH-REC(38) FC_2024), and the management of Luccahealth Medical Specialty Center. All patients aged 10 years and above who underwent transthoracic echocardiography between April 2024 and August 2024 (a total of 145) were included in the study. The exclusion criterion was patients whose weight and height were not recorded (4 patients). This was because their body mass index and body surface area could not be calculated, the latter essential for standardizing left ventricular mass index and left atrial volume index. Our cohort was therefore 141 patients, 132 of whom had LVGLS recorded. 

Age was categorized into three groups: 10-39 years, 40-69 years, and 70 years and above. Sex was categorized biologically as male or female. Body mass index (BMI) was classified as underweight-normal weight (≤ 24.9 kgm^-2^), overweight (25 - 29.9 kgm^-2^), and obese (≥ 30 kgm^-2^) [20]. Three systolic and diastolic blood pressure recordings were obtained, averaged, and classified using the American College of Cardiology/American Heart Association groups with some modification: systolic blood pressure was classified into three groups as normal, elevated + grade 1, and grade 2 hypertension, while diastolic blood pressure was grouped as normal, grade 1, and grade 2 hypertension [21].

Echocardiographic and strain data were obtained using the GE Vivid T9 V204 Ultrasound system, with Automatic Functional Imaging (AFI) version 3.0 (GE Healthcare, Chicago, IL, USA). The left atrial volume index (LAVi) was calculated using the biplane method (apical 4-chamber and apical 2-chamber views) and dichotomized as normal (≤ 34 ml/m^2^) or enlarged (> 34 ml/m^2^). Left ventricle geometry was identified as normal, concentric remodeling, concentric, or eccentric hypertrophy. This was based on evaluating left ventricular relative wall thickness (LVRWT) relative to left ventricular mass index (LVMI, in g/m^2^). E’ (e-prime) is the peak velocity of early mitral annular tissue velocity during diastole as measured by tissue Doppler imaging (TDI). In the apical 4-chamber (A4C) view, e’ was measured for both lateral and septal mitral annuli, and the average value was calculated. This average e’ was either normal (> 8.5 cms^-1^) or abnormal (≤ 8.5 cms^-1^), taking into consideration that abnormal septal e’ is < 7 cms^-1^ and the lateral < 10 cms^-1^. Average E/e’ (dimensionless), derived by dividing E velocity (from transmitral flow using pulsed wave Doppler) by average e’, was either normal (≤ 14) or abnormal (> 14). Peak tricuspid regurgitation velocity (TR), obtained by transtricuspid continuous-wave Doppler, was normal (≤ 2.8 ms^-1^) or abnormal (> 2.8 ms^-1^). LVEF was calculated using Simpson’s biplane method and classified as normal (≥ 50%) or abnormal (< 50%) [22]. LVGLS analysis was performed by a single operator, who obtained electrocardiogram-gated grayscale cineloops of the apical long, 4-, and 2-chamber views at approximately 60 frames per second. The region of interest was automatically defined as the myocardium between the endocardium and epicardium, and subsequently adjusted to accommodate full-thickness myocardium only. Offline strain analysis was performed using AFI 3.0 for each apical view. All segments were evaluated for quality. Where at least two segments were deficient, LVGLS was rejected; otherwise, the average LVGLS was accepted. Values were considered normal (< -18.0%), borderline (-18.0 - -16.0%), or abnormal (> -16.0%) [23]. 

This study did not account for variables such as medication and comorbidities, as most echocardiography requests are received from other health facilities and generally do not include such details. Indications for echocardiography were not considered for analysis.

Objectives

The specific objective was to determine the correlation between clinical risk factors and conventional echocardiographic/Doppler markers of diastolic and systolic dysfunction with LVGLS.

Data collection

A cardiologist retrieved consecutive reports of studies between April 2024 and August 2024. Reports where LVGLS was documented were selected. Clinical variables include age, gender, body mass index, and systolic and diastolic blood pressure. Echocardiographic data include left ventricular mass index, relative wall thickness, left atrial volume index, average e’, average E/e’, peak tricuspid regurgitation velocity, LVEF, and LVGLS. These were collected into a database. 

Statistical analysis

The data were captured and cleaned in Microsoft Excel version 16.57 (Microsoft Corporation, Redmond, WA, USA), and then exported to IBM SPSS Statistics version 27.0 (IBM Corp., Armonk, N.Y., USA) for further analysis. The data showed a non-normal distribution when a normality test was performed, as indicated by a normal Q-Q Plot, suggesting the use of a non-parametric test to determine the relationship between the study's variables. From the nonparametric test, descriptive statistics were extracted to show the data's dispersion around the mean for each study variable. Frequencies and percentages were also extracted to show the pattern displayed by the data. Further insights into the pairwise relationships were provided by the pairwise comparisons plot that visually represented the strength and significance of associations between all pairs of variables. A simple univariate linear regression analysis was conducted to examine the relationship between the dependent variable, LVGLS, and all other independent variables. The objective was to determine whether the independent variables can significantly predict LVGLS levels and to estimate the proportion of variance in LVGLS explained by the independent variables.

## Results

The descriptive characteristics of our patients are summarized in Table 1 and Table 2. 

**Table 1:** Descriptive statistics of categorical demographic/clinical data. BMI: body mass index; SBP: systolic blood pressure; DBP: diastolic blood pressure.

Variable	Categories	Number (%)
Age (years)	10 - 19	2 (1.4)
	20 - 29	5 (3.5)
	30 - 39	11 (7.8)
	40 - 49	31 (22.0)
	50 - 59	22 (15.6)
	60 - 69	36 (25.5)
	70 - 79	26 (18.4)
	≥80	8 (5.7)
Sex	Male	69 (48.9)
	Female	72 (51.1)
BMI (kgm^-2^)	Underweight	1 (0.7)
	Normal weight	15 (10.7)
	Overweight	48 (34.3)
	Obesity	76 (54.3)
SBP (mm Hg)	Normal	14 (10.3)
	Elevated	18 (13.2)
	Grade 1	24 (17.6)
	Grade 2	80 (58.8)
DBP (mm Hg)	Normal	46 (33.8)
	Grade 1	52 (38.2)
	Grade 2	38 (27.9)

**Table 2:** Summary statistics of clinical and echocardiographic risk factors associated with heart failure. BMI: body mass index; SBP: systolic blood pressure; DBP: diastolic blood pressure; LVMi: left ventricular mass index; LVRWT: left ventricular relative wall thickness; LAVi: left atrial volume index; TR: peak tricuspid regurgitation velocity; LVEF: left ventricular ejection fraction; LVGLS: left ventricular global longitudinal strain.

Variable	Median	Interquartile range	Mean ± standard deviation			Confidence interval (95%)
			Both sexes	Male	Female	
Age (years)	59.0	45.5 – 69.0	57.0 ±15.5	55.0	59.0	54.4 – 59.6
BMI (kgm^-2^)	30.5	27.3 – 35.1	31.5 ± 6.3	29.4	33.5	30.5 – 32.6
SBP (mm Hg)	143	130 – 158	145 ± 24	144	147	141 – 150
DBP (mm Hg)	83	73 – 92	83 ± 14	86	81	81 – 85
LVMi (gm^-2^)	69.15	57 – 87	75 ± 25	84	67	71 – 80
LVRWT	0.41	0.35 – 0.47	0.42 ±0.09	0.41	0.42	0.40 – 0.43
LAVi (ml/m^2^)	29.65	24 – 34	31 ± 11	31	31	29 – 33
e’ (cms^-1^)	8.95	7.4 – 11	9.3 ± 2.6	9.2	9.4	8.8 – 9.7
E/e’	7.80	6.6 – 10.0	8.5 ± 2.9	8.3	8.8	8.1 – 9.0
TR (ms^-1^)	2.7	2.2 – 2.9	2.6 ± 0.9	2.7	2.4	2.3 – 2.8
LVEF (%)	65	60 – 70	65 ± 7	64	66	63 – 66
LVGLS (%)	-17.00	-18.0 – -14.2	-16.4 ± 3.2	-16.0	-17.0	-17.0 – -15.9

**Figure 1:**
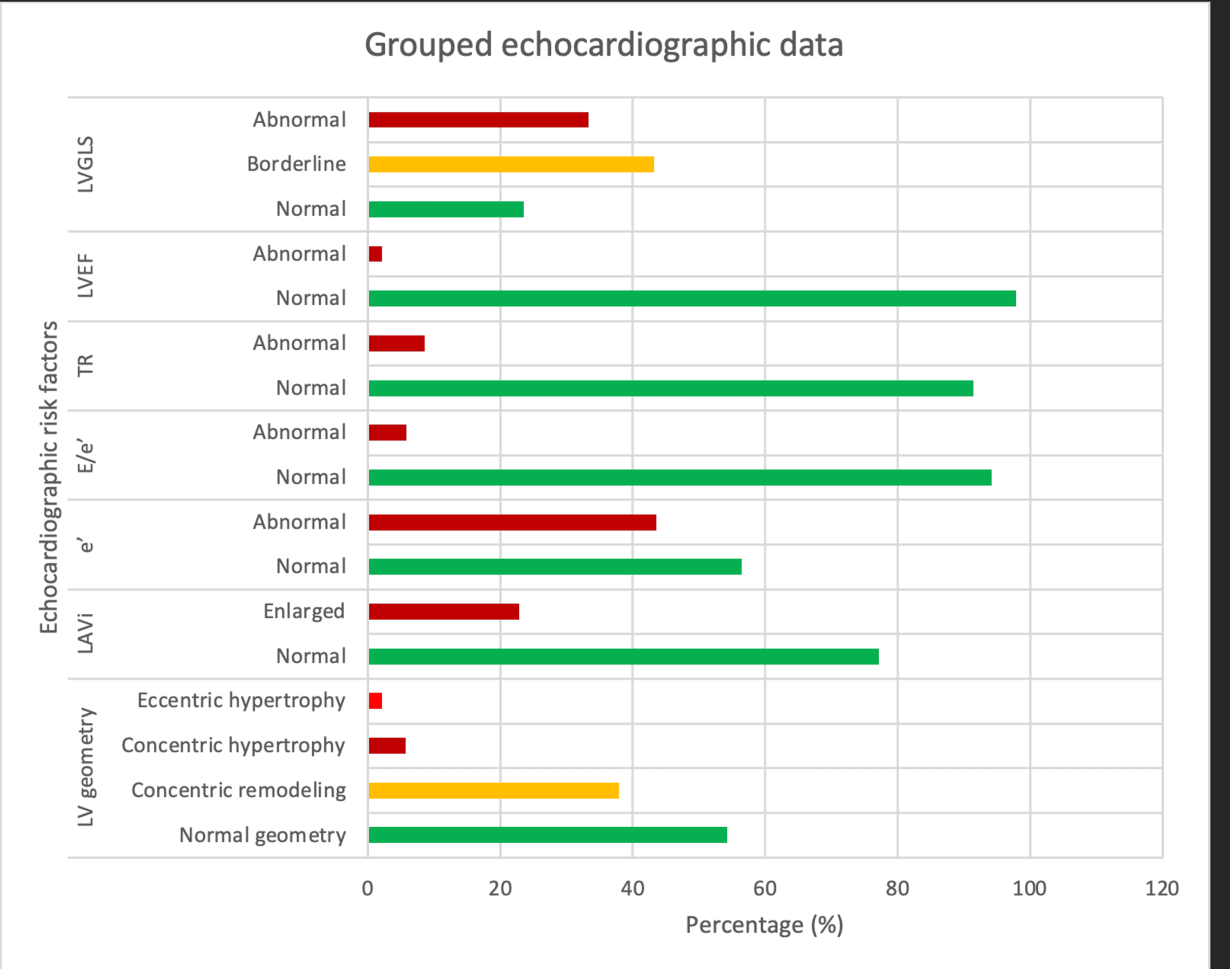
Grouped echocardiographic data derived from Table 3. LV geometry: left ventricular geometry; LAVi: left atrial volume index; TR: peak tricuspid regurgitation velocity; LVEF: left ventricular ejection fraction; LVGLS: left ventricular global longitudinal strain.

On echocardiographic parameters, 64 patients (45.7%) had abnormal left ventricular geometry (concentric remodeling 53 (37.9%), concentric hypertrophy 8 (5.7%), and eccentric hypertrophy 3 (2.1%), respectively), while 32 patients (22.9%) had an enlarged left atrium. Sixty-one (43.6%) patients, 8 (5.8%), and 12 (8.6%) had abnormal e’, E/e’, and tricuspid regurgitation, respectively. Only 3 (2.1%) patients had an abnormal LVEF. However, 57 (43.2%) and 44 (33.3%) had borderline and abnormal LVGLS, respectively (Table 3, Figure 1).

**Table 3:** Descriptive statistics of grouped echocardiographic data. LV geometry: left ventricular geometry; LAVi: left atrial volume index; TR: peak tricuspid regurgitation velocity; LVEF: left ventricular ejection fraction; LVGLS: left ventricular global longitudinal strain.

Variable	Groups	Number (%)
LV geometry	Normal geometry	76 (54.3)
	Concentric remodeling	53 (37.9)
	Concentric hypertrophy	8 (5.7)
	Eccentric hypertrophy	3 (2.1)
LAVi	Normal	108 (77.1)
	Enlarged	32 (22.9)
e’	Normal	79 (56.4)
	Abnormal	61 (43.6)
E/e’	Normal	129 (94.2)
	Abnormal	8 (5.8)
TR	Normal	128 (91.4)
	Abnormal	12 (8.6)
LVEF	Normal	137 (97.9)
	Abnormal	3 (2.1)
LVGLS	Normal	31 (23.5)
	Borderline	57 (43.2)
	Abnormal	44 (33.3)

Clinical variables, such as age, BMI, and SBP, when grouped, showed a statistically significant association with LVGLS, as indicated by crude p-values. Using adjusted p-values, age was the only clinical variable associated with LVGLS. We highlight that age had a very statistically significant association with LVGLS (p < 0.000). However, sex and diastolic blood pressure did not show this statistical association to LVLGS (Table 4).

**Table 4:** Relationship between clinical variables versus left ventricular global longitudinal strain. BMI: body mass index; SBP: systolic blood pressure; DBP: diastolic blood pressure; LVGLS: left ventricular global longitudinal strain. Pairwise comparison was used as a test of association, with a significance level set at 0.050. P-values are associated with Friedman's test statistic (using Friedman's 2-way analysis of variance). ^a^Significance values have been adjusted by the Bonferroni correction for multiple tests.

Variable		LVGLS			Statistical significance (p < 0.050)		Test statistic	
		Normal (%)	Borderline (%)	Abnormal (%)				
					Crude	Adjusted^a^		
Age	10 - 39	2 (1.5)	9 (6.8)	5 (3.8)	0.000	0.000	4.417	
	40 - 69	21 (15.9)	35 (26.5)	28 (21.2)				
	≥ 70	8 (6.1)	13 (9.8)	11 (8.3)				
Sex	Male	15 (11.4)	26 (19.7)	26 (19.7)	0.165	1.000	-1.181	
	Female	16 (12.1)	31 (23.5)	18 (13.6)				
BMI	Underweight/normal	8 (6.1)	6 (4.5)	2 (1.5)	0.001	0.080	2.750	
	Overweight	9 (6.8)	21 (15.9)	16 (12.1)				
	Obese	14 (10.6)	30 (22.7)	26 (19.7)				
SBP	Normal	8 (6.3)	4 (3.1)	2 (1.6)	0.004	0.280	2.431	
	Elevated/Grade 1	7 (5.5)	19 (15.0)	12 (9.4)				
	Grade 2	15 (11.8)	33 (26.0)	27 (21.3)				
DBP	Normal	14 (11.0)	21 (16.5)	7 (5.5)	0.870	1.000	-0.139	
	Grade 1	9 (7.1)	23 (18.1)	17 (13.4)				
	Grade 2	7 (5.5)	12 (9.4)	17 (13.4)				

Concentric and eccentric hypertrophy, under the variable LV geometry, were merged into “hypertrophy” for easier analysis. Echocardiographic variables such as LAVi, e’, E/e’, TR, and LVEF had a statistically significant association with LVGLS based on crude p-values, unlike LMVi (Table 5). It's notable that only 3 (2.3%) patients had abnormal LVEF; however, 57 (43.2%) and 41 (31.1%) who had normal LVEF also had borderline and abnormal LVGLS, respectively. Using adjusted p-values, E/e’ and LVEF were found to be statistically associated with LVGLS. This means that when the earlier variables are abnormal, LVGLS could decline. 

**Table 5:** Relationship between echocardiographic/Doppler variables versus left ventricular global longitudinal strain. LV geometry: left ventricular geometry; LAVi: left atrial volume index; TR: peak tricuspid regurgitation velocity; LVEF: left ventricular ejection fraction. Pairwise comparison was used as a test of association, with a significance level set at 0.050. P-values are associated with Friedman's test statistic (using Friedman's 2-way analysis of variance). ^a^Significance values have been adjusted by the Bonferroni correction for multiple tests.

Variable		LVGLS			Statistical significance (p < 0.050)		Test statistic
		Normal (%)	Borderline (%)	Abnormal (%)	Crude	Adjusted^a^	
LV geometry	Normal geometry	24 (18.2)	33 (25.0)	15 (11.4)	0.096	1.000	-1.417
	Concentric remodeling	7 (5.3)	23 (17.4)	20 (15.2)			
	Hypertrophy	0 (0.0)	1 (0.8)	9 (6.8)			
LAVi	Normal	25 (18.9)	44 (33.3)	28 (21.2)	0.004	0.252	-2.458
	Enlarged	6 (4.5)	13 (9.8)	16 (12.1)			
e’	Normal	25 (18.9)	32 (24.2)	16 (12.1)	0.012	0.819	-2.125
	Abnormal	6 (4.5)	25 (18.9)	28 (21.2)			
E/e’	Normal	31 (22.1)	51 (36.4)	50 (35.7)	0.000	0.001	-3.639
	Abnormal	0 (0.0)	4 (2.9)	4 (2.9)			
TR	Normal	7 (17.9)	15 (38.5)	6 (15.4)	0.002	0.133	-2.625
	Abnormal	2 (5.1)	7 (17.9)	2 (5.1)			
LVEF	Normal	31 (23.5)	57 (43.2)	41 (31.1)	0.000	0.000	-3.847
	Abnormal	0 (0.0)	0 (0.0)	3 (2.3)			

On regression analysis of clinical and echocardiographic/Doppler variables against LVGLS, BMI, SBP, DBP, LVMi, e', E/e', and LVEF demonstrated a significant relationship with LVGLS, with particular DBP, LVMi, e', E/e' showing a very significant relationship. Age, sex, RWT, LAVi, and TR did not correlate with LVGLS (Table 6, see Appendix A and Appendix B).

**Table 6:** Regression analysis between clinical and echocardiographic/Doppler variables with LVGLS. BMI: body mass index; SBP: systolic blood pressure; DBP: diastolic blood pressure; RWT: relative wall thickness; LVMi: left ventricular mass index; LAVi: left atrial volume index; TR: peak tricuspid regurgitation velocity; LVEF: left ventricular ejection fraction; LVGLS: left ventricular global longitudinal strain.

Independent variable		Sum of squares	df	Mean square	F	Statistical significance (p < 0.050)
Age	Regression	5.232	1	5.232	0.492	0.484
	Residual	1381.618	130	10.628		
	Total	1386.850	131			
Sex	Regression	17.824	1	17.824	1.693	0.196
	Residual	1369.026	130	10.531		
	Total	1386.850	131			
BMI	Regression	44.287	1	44.287	4.288	0.040
	Residual	1342.563	130	10.327		
	Total	1386.850	131			
SBP	Regression	59.569	1	59.569	6.578	0.012
	Residual	1131.995	125	9.056		
	Total	1191.565	126			
DBP	Regression	152.077	1	152.077	18.288	0.000
	Residual	1039.487	125	8.316		
	Total	1191.565	126			
RWT	Regression	70.676	1	70.676	6.981	0.009
	Residual	1316.174	130	10.124		
	Total	1386.850	131			
LVMi	Regression	194.188	1	194.188	21.166	0.000
	Residual	1192.662	130	9.174		
	Total	1386.850	131			
LAVi	Regression	72.758	1	72.758	7.198	0.008
	Residual	1314.092	130	10.108		
	Total	1386.850	131			
E’	Regression	225.882	1	225.882	25.293	0.000
	Residual	1160.968	130	8.931		
	Total	1386.850	131			
E/e’	Regression	141.584	1	141.584	14.563	0.000
	Residual	1244.408	128	9.722		
	Total	1385.992	129			
TR	Regression	5.717	1	5.717	0.888	0.352
	Residual	238.283	37	6.440		
	Total	244.000	38			
LVEF	Regression	116.981	1	116.981	11.976	0.001
	Residual	1269.869	130	9.768		
	Total	1386.850	131			

## Discussion

Cardiovascular diseases are the leading cause of death worldwide [1,24]. The prevalence and incidence of these diseases remain high in Ghana and many other countries [6]. Their etiology is variable, although they have a common confluence in the latter stages of their natural history, which is heart failure, and where the appropriate treatment, if it exists, but not applied, may invariably lead to premature death [25,26].

In Ghana, there is a significant burden of traditional cardiovascular risk factors, which has been documented [6,27]. Doku et al., in a meta-analysis of the prevalence of cardiovascular disease in Ghana, mainly using results from hospital-based studies, showed that prevalence was high, and modifiable risk factors included hypertension (pooled Odd’s ratio: 3.41; 95% CI: 1.75, 6.66) and diabetes mellitus (pooled Odd’s ratio: 2.79; 95% CI: 1.62, 4.81), amongst others [6]. This study also highlighted the need for standardized documentation of cardiovascular disease in Ghana. To our knowledge, population-based studies in this area of public health interest are lacking. Furthermore, studies documenting echocardiographic risk factors and preclinical markers of heart failure are few in Ghana [27,28]. Taking into consideration that overt heart failure is preceded by asymptomatic cardiac dysfunction, as demonstrated by Gidding et al. [29], detection and interventions to mitigate these risk factors are reasonable to prevent progression [10,30].

In our study, the burden of echocardiographic risk factors in a community clinic was notable (Table 3). These risk factors are mainly associated with heart failure with preserved systolic function, which currently has an increasing incidence and prevalence worldwide when compared to heart failure with reduced ejection fraction and comprises about half of all persons with heart failure, with the tendency to be the predominant form of heart failure in the near future [31-34].

The sample studied is not typically a “healthy” one, as evidenced by the high prevalence of obesity, elevated blood pressure, and abnormal echocardiographic parameters (Tables 1, 2). Referral bias may also be present, as patients referred to a cardiologist typically have a higher pre-test probability of cardiac disease. Age, sex, body mass index, blood pressure, and left ventricular mass index are known to affect LVGLS [35-37]. About 33% of our study population had abnormal LVGLS. Bendiab et al. found that 45.5% of their sample (N = 200), with normal LVEF, had abnormal LVGLS. Univariate analysis revealed that long-standing hypertension (>10 years), uncontrolled hypertension, left ventricular hypertrophy, diabetes, dyslipidemia, and renal failure were associated with this decline in LVGLS [38]. Some of these variables, such as blood pressure in the hypertensive range and left ventricular hypertrophy (LVMi), were documented in our patients and could, in part, account for the abnormal LVGLS in specific subgroups.

Determinants of LVGLS include clinical, hemodynamic, geometric, and myocardial tissue characteristics, as well as the quality of echocardiographic images [35]. Among the clinical parameters, LVGLS declines with age, as described by D’Elia et al. and other investigators [17,18,39]. In our study, we found a significant association between age groups when compared to the interpretation of LVGLS values (Table 4), but it did not correlate well with LVGLS (Table 6). D'Elia's population consisted of healthy individuals (those with known cardiac disease were excluded) [17]. In contrast, ours comprised a mixture of healthy individuals, those with risk factors for heart disease, and those with established heart disease. Dalen et al. found substantial differences in healthy individuals based on sex and age under 60 years in the HUNT (Helsetilsyn i Nord-Trøndelag or Nord-Trøndelag Health Study) study [39]. Takigiku et al., in the JUSTICE (Japanese Ultrasound Speckle Tracking of the Left Ventricle) study, demonstrated that the variability in the significance of these parameters may depend on the vendor of the speckle tracking echocardiography (STE) software [18]. Sex and specific age groups did have a significant relationship with LVGLS on General Electric and Philips systems, but not on Toshiba [18]. Irrespective of the vendor, an LVGLS < -16.0% is related to subclinical left ventricular systolic dysfunction [17]. However, in a recent study, Arrockiam et al. suggested new limits for LVGLS in healthy normal adults, with a mean of -17.0 (-20.3 - -13.7) [40], while LVGLS > -16.0 is indicative of left ventricular dysfunction [23].

Park et al. found significant sex differences in LVGLS (female subjects had better LVGLS than males −21.2 ± 2.2% vs. −19.5 ± 1.9%, p < 0.001) but not with age groups and LVGLS [37]. In contrast, a subgroup analysis of the MESA study also found no significant relationship between strain values and age, sex, or race [41]. This finding is consistent with our results, which revealed no significant correlation between age and sex when compared to LVGLS. Per our findings (Tables 4, 6), BMI (crude p-value) was associated with and correlated well with LVGLS (persons who are overweight or obese had lower LVGLS) [36,38]. We hypothesize that a higher BMI (assumed to be associated with a greater burden of adipose tissue) may affect the quality of images obtained in transthoracic echocardiography and, therefore, the quality of strain analysis [42,43].

Hemodynamic factors, including preload, contractility, afterload, and heart rate, affect LVGLS. Systolic and diastolic pressure are significantly associated with LVGLS, regardless of the STE software vendor. A physiologic rise in heart rate may lead to an increase in LVGLS [18,36,38]. A pathologic surge in heart rate, such as in sepsis, may lead to a fall in LVGLS [36]. Our study compared SBP and DBP categories with LVGLS and found an association between systolic blood pressure, but not diastolic blood pressure, and LVGLS categories, consistent with the findings of many other investigators; however, in regression analysis, both SBP and DBP were well correlated with LVGLS. 

Left ventricular geometry is a determinant of longitudinal strain. This may be explained by the distribution of wall stress based on Laplace’s law or other models [35,38]. Our analysis revealed an insignificant association between left ventricular geometry (as assessed by RWT and LVMi) and LVGLS. However, regression analysis showed a significant correlation with LVMi, but not with RWT, as independent variables differing somewhat from those of Park et al. found who found a significant relationship between left ventricular mass and LVGLS and left ventricular global longitudinal systolic strain rate; however, no significant relationship was found between left ventricular mass index and these two strain parameters in a healthy cohort [37]. We hypothesize that the difference in this observation may be due to the presence of individuals with cardiovascular risk factors and overt cardiac remodeling in our cohort. 

Per our analysis, LVGLS is significantly associated with and correlated well with LVEF. Delgado et al. similarly found good correlation between LVEF and LVGLS in their healthy control group, but not the group with acute coronary disease or ischemic heart failure [44]. LVGLS has been determined to enable the identification of persons with subclinical systolic dysfunction and offers incremental value when evaluating systolic function, especially when LVEF is preserved. This underscores the need to use LVGLS to risk-stratify patients, even if they have normal LVEF, enabling the prescription of optimal therapy for underlying cardiovascular risk factors, which may reduce morbidity and mortality [44-47]. Strain analysis may be performed using tissue Doppler imaging (TDI) or STE. Strain can be derived from strain rate and vice versa using calculus. TDI-derived e’ is the displacement rate of the mitral valve annulus. Integration of this rate with respect to time yields strain. Hence, it comes as no surprise that e’, in our study, has a statistically significant association and correlation with LVGLS, most likely because the measurement of e’ requires good alignment (parallel-to-longitudinal motion) to the mitral valve annulus in the apical 4-chamber view. This attenuates the angle-dependent limitation of TDI [16,47,48].

We found a statistical association between E/e’ and LVGLS. We highlight that e' and E/e’ correlate significantly with LVGLS. A lower e’ will result in a higher E/e’, both of which are established markers of diastolic dysfunction. Bendiab et al. found a statistical correlation between LAVi, LA filling pressure (derivable from E/e’), and PAP (derivable partly from TR) [38]. While our study similarly found good correlation between E/e' and LVGLS, the reason behind the discrepant results between LAVi and TR versus LVGLS is unclear to us. However, there is abundant information about the utility of these variables in evaluating left ventricular diastolic function and diagnosing heart failure with preserved ejection fraction. Hence, their routine assessment of patients is reasonable to identify patients at risk and to make the appropriate clinical interventions to reduce or curtail the incidence of heart failure [11,31,34,49].

Limitations of the study

This is a single-center, retrospective design that did not adjust for variables such as medications, comorbidities, etc., variables that could affect strain values. Vendor-specific software (GE) was used for strain analysis, which is known to exhibit some intervendor variability. Clinical endpoints such as follow-up outcomes were not assessed. A study with healthy participants will also be appropriate to establish baseline parameters.

## Conclusions

The burden of traditional risk factors for heart failure and structural/functional cardiac abnormalities, such as high blood pressure, obesity, left ventricular and atrial remodeling, e' prime, and abnormal LVGLS, is high among patients who presented to a community cardiologic clinic. Body mass index, systolic blood pressure, diastolic blood pressure, e’, E/e’, and LVEF independently correlated with LVGLS, a valuable parameter in evaluating LV systolic function, even when LVEF is normal, underscoring its role in risk stratification and early intervention for patients at risk of heart failure.

## Appendices

Appendix A 

**Table 7:** Model summary of regression. BMI: body mass index; SBP: systolic blood pressure; DBP: diastolic blood pressure; RWT: relative wall thickness; LVMi: left ventricular mass index; LAVi: left atrial volume index; TR: peak tricuspid regurgitation velocity; LVEF: left ventricular ejection fraction; LVGLS: left ventricular global longitudinal strain.

Dependent variable: LVGLS	R	R-square	Adjusted R-square	Standard error of the estimate
Predictor: (Age)	0.061	0.004	-0.004	3.2600
Predictor: (Sex)	0.113	0.013	0.005	3.2451
Predictor: (BMI)	0.179	0.032	0.024	3.2136
Predictor: (SBP)	0.224	0.050	0.042	3.0093
Predictor: (DBP)	0.357	0.128	0.121	2.8837
Predictor: (RWT)	0.226	0.051	0.044	3.1819
Predictor: (LVMi)	0.374	0.140	0.133	3.0289
Predictor: (LAVi)	0.229	0.052	0.045	3.1794
Predictor: (E')	0.404	0.163	0.156	2.9884
Predictor: (E/e')	0.320	0.102	0.095	3.1180
Predictor: (TR)	0.153	0.023	-0.003	2.5377
Predictor: (LVEF)	0.290	0.084	0.077	3.1254

Appendix B 

**Table 8:** Regresssion coefficients. BMI: body mass index; SBP: systolic blood pressure; DBP: diastolic blood pressure; RWT: relative wall thickness; LVMi: left ventricular mass index; LAVi: left atrial volume index; TR: peak tricuspid regurgitation velocity; LVEF: left ventricular ejection fraction; LVGLS: left ventricular global longitudinal strain.

Variable		Unstandardized coefficients B	Standard error	Standardized coefficients beta	t	Significance	Equation
1	Age	-17.106	1.114		-15.351	0.000	LVGLS = -17.106 + 0.013X
	Dependent variable: LVGLS	0.013	0.019	0.061	0.702	0.484	
2	Sex	-15.253	0.889		-17.153	0.000	LVGLS = -15.253 - 0.735X
	Dependent variable: LVGLS	-0.735	0.565	-0.113	-1.301	0.196	
3	BMI	-19.310	1.457		-13.257	0.000	LVGLS = -19.310 + 0.095X
	Dependent variable: LVGLS	0.095	0.046	0.179	2.071	0.040	
4	SBP	-20.432	1.590		-12.848	0.000	LVGLS = -20.432 + 0.028X
	Dependent variable: LVGLS	0.028	0.011	0.224	2.565	0.012	
5	DBP	-22.991	1.560		-14.740	0.000	LVGLS = -22.981 + 0.079X
	Dependent variable: LVGLS	0.079	0.018	0.357	4.276	0.000	
6	RWT	-19.889	1.368		-14.541	0.000	LVGLS = -19.889 + 8.385X
	Dependent variable: LVGLS	8.385	3.174	0.226	2.642	0.009	
7	LVMi	-19.966	0.829		-24.085	0.000	LVGLS = -19.966 + 0.048X
	Dependent variable: LVGLS	0.048	0.010	0.374	4.601	0.000	
8	LAVi	-18.384	0.807		-22.783	0.000	LVGLS = -18.384 + 0.066X
	Dependent variable: LVGLS	0.066	0.025	0.229	2.683	0.008	
9	E’	-11.674	0.966		-12.090	0.000	LVGLS = -11.674 - 0.504X
	Dependent variable: LVGLS	-0.504	0.100	-0.404	-5.029	0.000	
10	E/e’	-19.430	0.855		-22.736	0.000	LVGLS = -19.430 + 0.361X
	Dependent variable: LVGLS	0.361	0.095	0.320	3.816	0.000	
11	TR	-15.860	1.276		-12.431	0.000	LVGLS = -15.860 - 0.447X
	Dependent variable: LVGLS	-0.447	0.474	-0.153	-0.942	0.352	
12	LVEF	-7.565	2.553		-2.963	0.004	LVGLS = -7.565 - 0.136X
	Dependent variable: LVGLS	-0.136	0.039	-0.290	-3.461	0.001	
